# The UBC State Social Connection Scale: Factor Structure, Reliability, and Validity

**DOI:** 10.1177/19485506221132090

**Published:** 2022-11-01

**Authors:** Iris Lok, Elizabeth Dunn

**Affiliations:** 1 The University of British Columbia, Vancouver, Canada

**Keywords:** social connection, belonging, well-being, scale, measure

## Abstract

Social connection plays a central role in people’s everyday lives. Although researchers have traditionally focused on the benefits of experiencing an enduring sense of social connection, recent research has also begun to explore the contextual factors that shape momentary feeling of social connection. To date, however, no psychological scales have been developed to measure state social connection. To address this gap, we developed the 10-item UBC State Social Connection Scale (UBC-SSCS). In Study 1, we generated and refined our initial pool of items and confirmed our hypothesized factor structure in a large university sample. In Studies 2 to 3, we established several forms of validity. We provide foundational evidence that the UBC-SSCS is a reliable and valid instrument for assessing momentary feelings of social connection. Our exploratory findings also suggest that researchers can substantially increase their statistical power using state (vs. trait) measures to capture fluctuations in feelings of social connection.

Humans have a fundamental need for belonging. Over 25 years ago, Baumeister and Leary (1995) argued that people are innately motivated to form strong and positive social relationships, and achieve a sense of belonging that is characterized by a feeling of being socially connected to others. Since then, a large body of literature has examined the benefits of experiencing a stable and long-term sense of belonging (for a review, see Baumeister and Leary, 1995). In more recent years, researchers have shifted their attention to understanding the contextual factors that shape people’s ability to satisfy their belongingness needs. For example, Reis et al. (2000) found that people felt more socially connected when they engaged in social activities, such as talking about something meaningful and hanging out with others. Building on this work, Sandstrom and Dunn (2014) demonstrated that simply engaging in a brief conversation with a Starbucks cashier can lead to a greater sense of belonging. However, this sense of social connection that people derive from social interactions can be significantly undermined by the use of smartphones (e.g., Kushlev & Dunn, 2019, Dwyer et al., 2018). Across all of these studies, researchers have tried to capture momentary feelings of social connection by creating their own ad hoc measures or adapting trait measures of social connection. However, researchers are increasingly recognizing that utilizing measures with unknown psychometric properties can severely undermine their ability to make valid and reliable inferences about human behavior (e.g., Flake et al., 2017). Thus, the goal of the current research was to develop a valid and reliable instrument for measuring state social connection.

To date, researchers have developed several measures to capture trait levels of social connection. For example, the Social Connection Scale-Revised (SCS-R; Lee et al., 2001) includes 20 items that measure people’s feelings of closeness to the social world; it includes items that ask about specific social relationships (e.g., “My friends feel like family”), as well as items that tap into a more general sense of social connection (e.g., “I am in tune with the world.”). In a similar vein, the General Belongingness Scale (GBS; Malone et al., 2012) uses 12 items to assess the extent to which people have achieved a sense of belonging. Like the SCS-R, the scale contains a wide range of items that capture experiences of social connection in specific contexts (e.g., “I have close bonds with family and friends”) as well as more global contexts (e.g., “I feel connected with others.”). To capture the opposite of social connection, researchers have also measured loneliness using the 20-item University of California, Los Angeles (UCLA) Loneliness Scale (Russell, 1996), which measures how often people experience a sense of social isolation, using items, such as “How often do you feel that you lack companionship?” and “How often do you feel that there is no one you can turn to?”

In contrast, there are no existing scales that measure momentary feelings of social connection. As a result, researchers must adapt items from trait measures of social connection or develop their own items to measure state social connection. Although it is not uncommon for state and trait measures to be used interchangeably, researchers have argued that state and trait measures are meant to capture theoretically distinct constructs. Zuckerman (1983), for example, outlined the key features that differentiate trait and state scales. In particular, he argued that state—but not trait—measures should be sensitive to momentary changes in a given situation. Thus, a person’s state level of social connection should change following a brief social interaction, whereas their trait levels of social connection should remain relatively stable over time. In addition, researchers are increasingly recognizing the importance of validating survey instruments—even if they are simply adapting or re-purposing an existing scale (Flake & Fried, 2020). Because modifying the instructions and items on a scale can change its psychometric properties, adapted measures need to be re-evaluated to ensure that they are valid and reliable. As Furr (2011) puts it, “a modified scale is—to some degree—an ad hoc scale.”

In the current research, we developed a measure of state social connection. To create our scale while maximizing consistency with past work, we adapted items from existing measures of trait social connection. We then assessed the structure of the UBC State Social Connection Scale (UBC-SSCS) using exploratory and confirmatory factor analysis (Study 1) and evaluated the extent to which the scale demonstrated several forms of validity (Studies 2 and 3). The materials, data, and code for our studies are available at https://tinyurl.com/9kr6j9b6.

## Study 1

The goal of Study 1 was to develop and refine a measure of state social connection. We created an initial pool of 11 items that were adapted from existing measures of trait social connection, including the SCS-R (Lee et al., 2001) and the GBS (Malone et al., 2012 see Table 1 for list of items). We selected items using two rules: (1) the items should capture a general feeling of connection that is not specific to any situation, individual, or group, (2) the items should capture a wide range of subjective experiences with some of the items being endorsed more frequently (“easy” items), and other items being endorsed less frequently (“difficult” items). Then, we created a set of instructions for completing the items. Because the scale is meant to capture momentary feelings of social connection, our instructions first ask participants to think about how they felt during a particular window of time (e.g., in the last 15 min). Then, participants are asked to indicate how much each item describes how they felt. Finally, we collected data from a large sample of participants and conducted factor analyses to evaluate the structure of our scale.

**Table 1.** UBC State Social Connection Scale: Initial Item Pool.

1. I felt distant from people (R)
2. I didn’t feel related to most people (R)
3. I felt like an outsider (R)
4. I felt like I was able to connect with other people
5. I felt disconnected from the world around me (R)
6. I felt close to people
7. I saw people as friendly and approachable
8. I was in tune with the world. (item excluded from the final 10-item scale)
9. I felt accepted by others
10. I had a sense of belonging
11. I felt a strong bond with other people

*Note.* Participants read the following prompt: “Please think about how you felt during [time frame]. To what extent do each of the following statements describe how you felt?” Participants then indicated the extent to which they agreed or disagreed with each statement on a 7-point Likert-type scale (1 *= Strongly disagree*, 4 = *Neither agree nor disagree*, and 7 = *Strongly agree*).

### Method

#### Participants and Procedures

We planned to collect as much data as possible from a department-wide pretesting survey. Based on past experience with this recruitment strategy, we expected to include at least 1,000 participants in our sample. In total, 1,031 undergraduate students enrolled in the University of British Columbia (UBC; see Table 2 for demographics) completed the UBC-SSCS in exchange for course credit.

**Table 2.** Study 1: Demographics.

Mean age (*SD*)	21.2 (3.7)
Gender	
Female	76.3%
Male	23.1%
Trans	0.1%
Other	0.5%
Ethnicity	
East Asian	45.4%
European	21.1%
South Asian	11.4%
South East Asian	9.5%
Middle Eastern	3.4%
African	1.3%
Hispanic	1.3%
First Nations	0.1%
Other	6.6%

To measure state social connection, we presented participants with the following instructions: “Please think about how you felt today. To what extent do each of the following statements describe how you felt?” Then, we asked participants to indicate the extent to which they agreed with each item on a scale from 1 (*Strongly disagree*) to 7 (*Strongly agree*).

### Results

We randomly split our sample into two halves and conducted exploratory factor analysis (EFA) on one half (*N* = 515), and confirmatory factor analysis (CFA) on the other half (*N* = 516). This approach allowed us to test whether our hypothesized factor structure is sustained in each independent sample.

#### Exploratory Factor Analysis

##### Factor Extraction

We used scree plots and parallel analysis to determine the number of factors to extract from our model. The scree plot pointed to a one-factor solution, whereas parallel analysis pointed to the possibility of a two-factor solution. Thus, we conducted EFAs to test a one- and two-factor solution.

##### One-Factor Solution

We tested a one-factor solution using least squares extraction with the *psych* package in R (see Table 3). The standardized factor loadings ranged from .64 to .81; in other words, the latent factor explained at least 41% of the observed variance for each item. Overall, the one-factor solution accounted for 53% of the observed variance across the 11 items. We also examined the correlation between the item residuals (see Table 4). On average, the reverse-coded items were associated with larger residuals, suggesting that participants may be responding to those items differently.

**Table 3.** One-Factor EFA Solution.

Item	Standardized loading	*M* (*SD*)
1. I felt distant from people (R)	.72	4.08 (1.64)
2. I didn’t feel related to most people (R)	.71	4.52 (1.49)
3. I felt like an outsider (R)	.72	4.62 (1.62)
4. I felt like I was able to connect with other people	.77	4.87 (1.37)
5. I felt disconnected from the world around me (R)	.67	4.21 (1.62)
6. I felt close to people	.81	4.77 (1.48)
7. I saw people as friendly and approachable	.70	4.95 (1.33)
8. I was in tune with the world	.64	4.47 (1.34)
9. I felt accepted by others	.75	5.10 (1.29)
10. I had a sense of belonging	.78	4.83 (1.43)
11. I felt a strong bond with other people	.76	4.85 (1.45)

**Table 4.** One-Factor EFA Solution Residual Correlation Matrix.

Item	1	2	3	4	5	6	7	8	9	10	11
1. I felt distant from people (R)	–	.13	.08	−.07	.40	−.03	−.12	−.04	−.18	−.16	−.12
2. I didn’t feel related to most people (R)		–	.32	−.11	−.001	−.07	−.05	−.10	−.12	−.06	−.05
3. I felt like an outsider (R)			–	−.17	.01	−.17	−.03	−.07	.08	.03	−.17
4. I felt like I was able to connect with other people				–	−.03	.09	.10	.01	.02	.02	.17
5. I felt disconnected from the world around me (R)					–	−.14	−.14	.27	−.18	−.14	−.23
6. I felt close to people						–	.02	−.02	.01	.07	.24
7. I saw people as friendly and approachable							–	.02	.15	.04	.04
8. I was in tune with the world								–	−.04	.01	−.06
9. I felt accepted by others									–	.17	.05
10. I had a sense of belonging										–	.06
11. I felt a strong bond with other people											–
*Mean residual correlation* (*in absolute value*)	.13	.10	.11	.08	.15	.09	.07	.06	.10	.08	.12

##### Two-Factor Solution

We tested a two-factor solution using least squares extraction and oblimin rotation using the *psych* package in R (see Table 5). The first factor tapped into social connection; the six items that loaded strongly onto this factor were all positively worded statements that referred to feeling a strong sense of connection. The second factor tapped into social *dis*connection; the four items that strongly loaded onto this factor were all negatively worded statements that referred to a lack of connection. Of course, because these four items were all reverse-coded, the second factor might also reflect common-method variance (e.g., participants responding to reverse-coded items in a systematic way). Nevertheless, there was a strong positive correlation between the two factors (*r* = .74), suggesting that the two factors could be treated as a single factor. The two-factor solution also revealed a problematic item that did not strongly load onto either factor (“I was in tune with the world.”). Thus, the item might not be measuring social connection in the way that we intended. Indeed, the problematic item was the only item that did not make any reference to other people or the feeling of being connected.

**Table 5.** Two-Factor EFA Solution.

Item	Loadings		*M* (*SD*)
	Factor 1	Factor 2	
I felt distant from people (R)	.03	**.77**	4.00 (1.63)
I didn’t feel related to most people (R)	.28	**.48**	4.43 (1.57)
I felt like an outsider (R)	.31	**.45**	4.57 (1.64)
I felt like I was able to connect with other people	**.72**	.08	4.83 (1.44)
I felt disconnected from the world around me (R)	−.07	**.83**	4.21 (1.63)
I felt close to people	**.78**	.07	4.71 (1.47)
I saw people as friendly and approachable	**.71**	.02	4.97 (1.28)
I was in tune with the world^a^	.30	.38	4.37 (1.37)
I felt accepted by others	**.82**	−.04	5.07 (1.28)
I had a sense of belonging	**.77**	.05	4.94 (1.42)
I felt a strong bond with other people	**.87**	−.08	4.70 (1.53)

*Note.* Standardized factor loadings > .40 are bolded.

a Indicates items with weak loadings on each factor.

**Figure 1.**
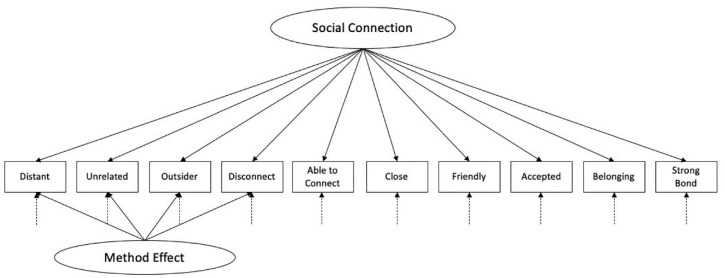
Final Hypothesized Factor Model.

Based on our results, we retained a one-factor solution with all items loading onto a single factor representing state social connection. To account for common-method variance, we cross-loaded the four reverse-coded items onto a second factor representing method effects. Finally, we removed the problematic item from our scale. Figure 1 illustrates our final hypothesized factor model.

#### Confirmatory Factor Analysis

We tested our final hypothesized factor model using the lavaan package in R. We accounted for any missing data using maximum likelihood estimation. To assess model fit, we assessed the following fit indices: chi-square test of exact fit, comparative fit index (CFI), Tucker–Lewis index (TLI), root mean square error of approximation (RMSEA), and squared root mean residual (SRMR).

Table 6 presents the results of our analysis. Overall, the model fit the data well based on the CFI (0.97), TLI (0.96), RMSEA (0.08), and SRMR (0.02), although it did not satisfy the χ^2^ test of exact fit (which is expected in large samples; see Bandalos, 2018). In addition, we did not find any large residual correlations between any of the items (all *r*s < |,07|). The social connection factor also demonstrated high levels of reliability (α = .92; ω = .93).

**Table 6.** Final Hypothesized Factor Model CFA Solution.

Item	Standardized factor loading	
	Social connection	Method effect
1. I felt distant from people (R)	.64	.53
2. I didn’t feel related to most people (R)	.68	.36
3. I felt like an outsider (R)	.68	.33
4. I felt like I was able to connect with other people	.79	–
5. I felt disconnected from the world around me (R)	.56	.46
6. I felt close to people	.85	–
7. I saw people as friendly and approachable	.60	–
8. I felt accepted by others	.80	–
9. I had a sense of belonging	.83	–
10. I felt a strong bond with other people	.81	–

#### Discussion

In Study 1, we confirmed our hypothesized factor structure using a large sample of participants. Each item loaded strongly onto the latent factor representing state social connection (with the underlying construct explaining at least ≈30% of the observed variance for each item). In addition, our items did not seem to suffer from ceiling or floor effects based on their means and standard deviations—which tended to fall toward the middle of the scale (see Tables 3 and 5). In Studies 2 and 3, we sought to establish the validity of the UBC-SSCS.

## Study 2

In Study 2, we tested whether the UBC-SSCS was sensitive to experimental manipulations that were meant to influence momentary levels of social connection. To address this question, we randomly assigned participants to write about the last experience in which they spent time either alone or with others. Because social connection is characterized by feeling accepted, feeling close to others, as well as a sense of belonging, we expected participants to report higher levels of social connection when think about spending time with other people (vs. alone).

In addition, we assessed the relationship between the UBC-SSCS and other theoretically relevant constructs, including mood, satisfaction with life, and loneliness. Because social connection is associated with both affective and cognitive dimensions of subjective well-being (see Diener & Seligman, 2002), we expected state social connection to be associated with higher levels of positive mood and life satisfaction and lower levels of negative mood and loneliness.

Finally, we examined whether state and trait social connection are related but distinct constructs by looking at the correlation between trait and state levels of social connection. We also compared the correlation between state–state, trait–trait, and state–trait measures. Based on Zuckerman’s (1983) criteria for distinguishing state and trait measures, we expected state measures of positive and negative mood to be more strongly associated with state—rather than trait—levels of social connection. In contrast, we expected trait measures of life satisfaction, loneliness, and extraversion to be more strongly associated with trait—rather than state—levels of social connection.

### Method

#### Overview

We measured participants’ trait levels of social connection, satisfaction with life, loneliness, and extraversion. Then, we randomly assigned them to think about a time when they were either alone or with others, and asked them to report their state levels of social connection and mood in that situation.

#### Participants and Procedures

Because we expected to observe a relatively large difference between conditions, as well as strong correlations between our measures, we recruited 202 participants from Prolific who completed the study in exchange for 1.50 Canadian dollars (see Table 7 for demographics). Based on a sensitivity analysis conducted in G*Power 3.1, this sample size enabled us to detect a medium-to-large effect size (*d* = 0.40) and small correlation (*r* = .19) with 80% Power (two-tailed).

**Table 7.** Study 2: Demographics.

Mean age (*SD*)	29.01 (8.88)
Gender	
Male	65.84%
Female	33.17%
Nonbinary/other	0.99%
Ethnicity	
White	80.20%
Asian	6.44%
Black	1.49%
Mixed	4.46%
Other	7.43%

Upon providing consent, participants completed all our trait measures:

##### Trait Social Connection

To measure trait social connection, we presented participants with 20 statements reflecting the various ways in which people can view themselves (e.g., “I fit in well in new situations”) using the SCS-R (Lee et al., 2001; α = .95). We then asked them to rate the extent to which they agree or disagree with each statement on a scale from 1 (*Strongly disagree*) to 6 (*Strongly agree*).

##### Life Satisfaction

To measure life satisfaction, we asked participants to rate their satisfaction with life using the five-item Satisfaction with Life Scale (Diener et al., 1985; α = .90). Participants rated their agreement with statements like, “In most ways, my life is close to my ideal” and “I am satisfied with my life” on a scale from 1 (*Strongly disagree*) to 7 (*Strongly agree*).

##### Loneliness

To measure loneliness, we asked participants to report how often they feel lonely using the 20-item UCLA Loneliness Scale Version 3 (Russell, 1996; α = .94). Participants responded to statements like, “How often do you feel alone?” and “How often do you feel that your relationships with others are not meaningful?” on a scale from 1 (*Never*) to 4 (*Always*).

##### Extraversion

To measure extraversion, we asked participants to rate the extent to which a number of characteristics (e.g., “Is outgoing, sociable”) apply to them on a scale from 1 (*Disagree strongly*) to 5 (*Agree strongly*), using the six-item extraversion subscale from the BFI-2-S (Soto & John, 2017; α = .79).

Then, participants were randomly assigned to one of two conditions. In the *alone* condition, participants were told to describe the most recent occasion in which they spent time alone. In the *together* condition, participants were told to describe the most recent occasion in which they spent time with people they cared about. Afterwards, participants reported their state level of social connection and mood during the experience they had just described. Finally, they reported their age, gender, and ethnicity.

##### State Social Connection

To measure state social connection, we asked participants to think about how they felt during the experience they had just described, and to report the extent to which they agreed with our 10 items on a scale from 1 (*Strongly disagree*) to 7 (*Strongly agree*; α = .95).

##### Mood

To measure mood, we asked participants to indicate how often they felt a range of positive emotions (e.g., “Good”) and negative emotions (e.g., “Sad”) during the experience they described on a scale from 1 (*Very rarely or never*) to 5 (*Very often or always*) using 12 items from the Scale of Positive and Negative Experiences (Diener et al., 2010; α_Positive Affect_ = .95, α_Negative Affect_ = .93).

### Results

To assess whether the UBC-SSCS responded to our experimental manipulation, we compared participants’ ratings of state social connection in the alone versus together condition. As expected, participants in the together condition reported feeling more socially connected while thinking about their experience (*M* = 5.35, *SD* = 1.24) compared with those in the alone condition (*M* = 3.54, *SD* = 1.29), *t*(199.95) = −10.14, *p* < .001, *d* = 1.42.

Next, we assessed a broader form of construct validity by looking at the zero-order correlations for state social connection, trait social connection, positive and negative mood, life satisfaction, loneliness, and extraversion (see Table 8). Across both conditions, feeling socially connected at the state level was strongly associated with higher levels of positive mood and life satisfaction, and lower levels of negative mood and loneliness.

**Table 8.** Study 2: Correlation Across Variables.

Measure	1	2	3	4	5	6	7
1. State social connection	–	.54*	.69*	−.60*	.30*	−.52*	.29*
2. Trait social connection		–	.39*	−.35*	.52*	−.87*	.62*
3. State positive mood			–	−.78*	.37*	−.43*	.23*
4. State negative mood				–	−.27*	.40*	−.12
5. Trait life satisfaction					–	−.57*	.40*
6. Trait loneliness						–	−.56*
7. Trait extraversion							–
*M*	4.44	3.58	3.48	2.11	3.76	2.49	2.69
*SD*	1.56	0.92	1.05	1.05	1.39	0.57	0.79

* *p* < .001.

We also found a moderate positive correlation between trait and state levels of social connection (*r* = .54). Consistent with Zuckerman’s (1983) theorizing, state measures of positive and negative mood were more strongly correlated with state social connection than with trait social connection. Likewise, trait measures of life satisfaction, loneliness, and extraversion were more strongly correlated with trait social connection than with state social connection. Together, these findings provide preliminary evidence that state and trait social connection are related but distinct constructs (for significance tests, see Table 9).

**Table 9.** Study 2: Difference Between Paired Correlations.

Measure	Correlation (*r*)		*t*	*p*
	State social connection	Trait social connection		
State positive affect	.69	.39	6.04	<.001
State negative affect	−.60	−.35	−4.50	<.001
Trait life satisfaction	.30	.52	−3.68	<.001
Trait loneliness	−.52	−.87	9.99	<.001
Trait extraversion	.29	.62	−6.03	<.001

### Discussion

In Study 2, we found some preliminary evidence of construct validity. First, we showed that participants who wrote about spending time with others (vs. by themselves) reported higher levels of state social connection while thinking about that experience. Second, we demonstrated that state social connection was associated with higher levels of positive mood and life satisfaction, and lower levels of negative mood and loneliness. Finally, participants reported state levels of social connection that were moderately correlated with their trait levels of social connection, which were reported at the beginning of the study; this provides some initial evidence that trait and state social connection are related but distinct constructs. In Study 3, we built on this initial work by examining whether the UBC-SSCS exhibits known-groups validity and discriminant validity.

## Study 3

In Study 3, our first goal was to assess whether the UBC-SSCS can detect differences in momentary feelings of social connection between existing groups (establishing known-groups validity). Instead of using a recall task to manipulate participants’ state levels of social connection, we selectively recruited participants who were either by themselves or socializing with others. Following our previous findings, we predicted that solo (vs. socializing) participants would report lower levels of state social connection.

Our second goal was to show that the UBC-SSCS is more sensitive to variations in people’s momentary experiences compared with standard measures of trait social connection (establishing discriminant validity). Because being temporarily deprived of social contact (e.g., being alone) should have a bigger impact on people’s momentary—rather than enduring—feelings of social connection, we expected solo participants to report lower relative levels of state (vs. trait) social connection. Likewise, we expected socializing participants to report higher relative levels of state (vs. trait) social connection.

### Method

#### Overview

We invited people who were spending time at UBC to report their trait and state levels of social connection. To establish known groups and discriminant validity, our research team selectively recruited participants who were by themselves or socializing with others. The pre-registration, data and materials for this study can be found at https://tinyurl.com/9kr6j9b6.

#### Participants

A sample of 1,260 eligible participants (28.4% male, 68.5% female, 1.6% non-binary, and 1.5% preferred not to disclose) completed the study for a chance to win a $500 Canadian dollar gift card. The mean age was 20.66 years old (*SD* = 3.43, range = 13–58).

#### Procedures and Measures

Research assistants approached individuals at the UBC campus who appeared to be either spending time by themselves (*solo* group) or socializing with others (*socializing* group). Upon providing consent, participants were asked to complete measures of trait and state social connection. Because the first measure of social connection that participants complete may shape the way they respond to the other measure, we randomly assigned participants to complete a measure of trait social connection followed by a measure of state social connection (*trait-first* condition) or to complete the same measures in the opposite order (*state-first* condition). Next, participants reported whether they were currently socializing with other people, and whether they were currently spending time with anyone else. Finally, participants reported their gender and age.

#### Trait Social Connection

To measure trait social connection, we asked participants to report their agreement with twelve statements from the GBS (Malone et al., 2012α = .91) on a scale of 1 (*Strongly disagree*) to 7 (*Strongly agree*).

#### State Social Connection

To measure state social connection, we asked participants to complete the UBC-SSCS with regard to their current feelings (α = .90).

### Results

#### Pre-Registered Exclusion Criteria

We applied all exclusions based on our preregistered criteria (Table 10). Because the goal of the current study was to compare the UBC-SSCS against a trait measure of social connection, we excluded participants who missed any social connections items on the survey. To ensure that we correctly categorized participants in each known group, we only included participants in the solo group if (1) a research assistant observed that they were alone and (2) the participant self-reported that they were not socializing or spending time with others. Likewise, we only included participants in the socializing group if observer and self-report ratings converged.

**Table 10.** Study 3: Participant Exclusions.

Number of participants missing items on measures of social connection	136
Number of participants in the socializing group who indicated that they were not socializing with others in the survey	54
Number of participants in the solo group who indicated that they were socializing or spending time with others in the survey	319
Number of participants who did not report whether they were socializing or spending time with others in the survey	52
Total number of participants	1,821
Total number of eligible participants	1,260

We originally planned to recruit 139 participants per cell to detect a small-to-medium difference between any two cells with 80% power (one-tailed). However, we recruited a large sample of participants to ensure we would meet our goal after applying our exclusion criteria. We used the full sample of participants (*N* = 1,260) in our reported analyses to maximize power. However, our confirmatory results are identical when we randomly sample 139 participants per cell (tinyurl.com/y8jhy3h4).

#### Preregistered Analytic Strategy

Following our pre-registration, we used two-way interactive analysis of variance (ANOVA) models and planned contrasts to conduct our confirmatory analyses. In line with recent recommendations, all directional hypotheses were examined using one-tailed tests (Lakens, 2016). Only the first social connection scale (trait or state) that participants completed was analyzed in our confirmatory tests.

#### Preregistered Analyses

##### Known-Group Validity

To establish known-group validity, we examined whether the state social connection scale could detect differences between participants who were spending time on their own and those who were socializing. As expected, solo participants reported significantly lower levels of state social connection (*M* = 4.51, *SD* = 1.05) than those who were socializing (*M* = 4.92, *SD* = 0.93), *t*(1,256) = 4.75, *p* < .001, *d* = 0.41.

##### Discriminant Validity

To establish discriminant validity, we compared state and trait social connection ratings within each known group. Because we were primarily interested in comparing relative—rather than absolute—ratings of social connection, we preregistered that we would use standardized scores for this set of analyses. We found mixed evidence of discriminant validity. As expected, solo participants reported relatively lower levels of state social connection (*M* = −0.30, *SD* = 1.07) compared with trait social connection (*M* = −0.11, *SD* = 1.10), *t*(1,256) = −1.84, *p* = .03, *d* = 0.18. In contrast, there was no significant difference in relative ratings of state social connection (*M* = 0.11, *SD* = 0.95) and trait social connection (*M* = 0.05, *SD* = 0.95) among socializing participants, *t*(1,256) = 0.98, *p* = .16, *d* = 0.06.

#### Exploratory Analyses

##### Solo versus Socializing Participants on Trait Social Connection

**Figure 2.**
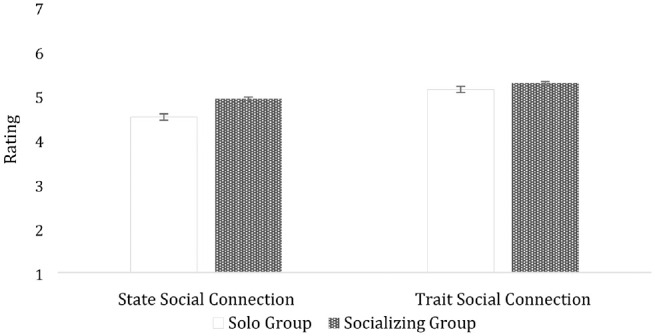
Solo Versus Socializing Group: State and Trait Social Connection Ratings.

On an exploratory basis, we used the same analytic approach to compare solo (vs. socializing) participants on the trait social connection scale. Solo participants experienced marginally lower levels of trait social connection (*M* = 5.13, *SD* = 1.00) than socializing participants (*M* = 5.27, *SD* = 0.87), *t*(1,256) = 1.72, *p* = .08, *d* = 0.15 (two-tailed). However, this effect size was almost three times smaller than the difference between solo versus socializing participants on the UBC-SSCS. Indeed, when we entered group (solo vs. socializing) and type of measure (state vs. into an 2 × 2 interactive ANOVA, there was a significant Group × Measure interaction, *t*(1,256) = 2.25, *p* = .02 (two-tailed; see Figure 2).

##### Within-Subject Data

Although all participants completed the state and trait measures of social connection, we only used the first set of ratings in our confirmatory analyses. Harnessing all the available data, we repeated the same analyses using a two-way mixed ANOVA (with two-tailed tests). We entered group (solo vs. socializing) as a between-subjects factor, and type of measure (state vs. trait) as a within-subject factor, to predict social connection ratings. We found supporting evidence for known-groups validity. Compared with those who were by themselves, socializing participants were more socially connected at the state, *t*(1,258) = 5.67, *p* < .001, *d* = 0.34, and trait level, *t*(1,258) = 2.93, *p* = .003, *d* = 0.18. However, the effect size was almost twice as large for state (vs. trait) social connection, creating a significant Group × Measure-type interaction, *t*(1,258) = 5.51, *p* < .001.

There was also evidence of discriminant validity. Solo participants reported relatively lower levels of state social connection (*M* = −0.25, *SD* = 1.10) than trait social connection (*M* = −0.13, *SD* = 1.08), *t*(1,258) = −4.20, *p* < .001, *d* = 0.11. Similarly, socializing participants reported relatively higher levels of state social connection (*M* = 0.10, *SD* = 0.94) than trait social connection (*M* = 0.05, *SD* = 0.96), *t*(1,258) = 2.61, *p* = .009, *d* = 0.05.

##### Measurement Invariance

Our sample size enabled us to assess whether the UBC-SSCS performs differently for male and female participants. Overall, we found tentative evidence for configural, metric, and scalar invariance (see Online Supplemental Material).

### Discussion

Study 3 provided the first evidence of known-groups validity for the UBC-SSCS. Compared with participants who were socializing with others, solo participants experienced lower levels of state social connection. Solo (vs. socializing) participants also reported lower levels of trait social connection in our exploratory analyses; however, the difference between solo and socializing participants was almost three times smaller at the trait (vs. state) level.

We also found some evidence for discriminant validity. Solo participants reported lower levels of state (vs. trait) social connection in both our between-subjects (preregistered) and within-subjects (exploratory) tests. There was no significant difference in relative levels of state and trait social connection among socializing participants in our between-subjects (preregistered) tests, but this difference was significant using within-subjects (exploratory) tests. This suggests that people may reliably experience reduced social connection when they are alone, but that the level of social connection they experience when socializing may depend on important features of the situation (e.g., type of relationship partner).

## General Discussion

Across four studies, we developed and validated the UBC-SSCS. In Study 1, we created our initial pool of items by adapting existing measures of trait social connection, facilitating comparisons of state and trait measures of social connection in later studies. Then, we refined our items and confirmed our hypothesized factor structure in a large sample of undergraduate students. In Study 2, we established that the scale was psychometrically sound—correlating with theoretically relevant constructs and discriminating between participants who thought about a time they were alone versus with other people. In Study 3, we provide initial evidence of known-groups validity and discriminant validity. Notably, our exploratory analyses revealed that the difference between participants who were alone versus socializing with others was almost three times stronger when social connection was measured at the state (*d* = 0.41) rather than trait level (*d* = 0.15). This means that researchers would only need 95 participants per cell to detect a difference between solo versus socializing participants using the UBC-SSCS, whereas they would need 699 participants per cell to achieve the same power using a comparable trait measure. Or, given 100 participants per cell, researchers would have approximately 80% power using the UBC-SSCS versus approximately 20% power using a trait measure of social connection. Thus, although trait social connection scales can capture differences between distinct groups, trait measures may severely limit researchers’ ability to detect effects in smaller samples.

An important limitation of this research is that the scale may not be suitable for all cultures. Although the scale was shown to be reliable and valid among undergraduate students in Canada (Studies 1 and 3) and adults in the United States and the United Kingdom (Study 2), it is unclear whether the scale would demonstrate similar psychometric properties in populations outside Europe and North America. Thus, it would be worthwhile to test the scale in other cultural contexts.

Across disciplines from psychology to public health, researchers are increasingly recognizing the importance of social connection in people’s lives. In one meta-analysis, for example, researchers argued that being socially disconnected was equivalent to smoking 15 cigarettes a day (Holt-Lunstad et al., 2010). Thus, to combat what is increasingly called the “loneliness epidemic,” an important first step will be to identify the contextual factors that promote a sense of social connection. For example, social psychologists might draw from past research and test whether introducing a “no technology” policy in the first few minutes before class would help students feel more socially connected during class. The UBC-SSCS contributes to this endeavor by offering a valid and reliable instrument for evaluating whether interventions—such as a “no technology” policy—can lead to meaningful fluctuations in people’s feelings of social connection.

## Supplemental Material

sj-docx-1-spp-10.1177_19485506221132090 – Supplemental material for The UBC State Social Connection Scale: Factor Structure, Reliability, and Validity Click here for additional data file. Supplemental material, sj-docx-1-spp-10.1177_19485506221132090 for The UBC State Social Connection Scale: Factor Structure, Reliability, and Validity by Iris Lok and Elizabeth Dunn in Social Psychological and Personality Science
