# Supplementary material for: The UBC State Social Connection Scale: Factor Structure, Reliability, and Validity
Source: Soc Psychol Personal Sci. 2022 Nov 1;14(7):835–44. doi: 10.1177/19485506221132090 (PMC10396795; doi:10.1177/19485506221132090)
Supplement: sj-docx-1-spp-10.1177_19485506221132090 – Supplemental material for The UBC State Social Connection Scale: Factor Structure, Reliability, and Validity [file sj-docx-1-spp-10.1177_19485506221132090.docx]

**Study 3: Measurement Invariance**

Our sample size enabled us to assess whether the UBC State Social Connection Scale (UBC-SSCS) performs differently for male (28.4% of the sample, n = 358) and female participants (68.5% of the sample, n = 863). To start, we tested two CFA models with unconstrained factor loadings and intercepts for male and female participants respectively. The model demonstrated good fit for each group (Table 1) and thus, we proceeded to test for measurement invariance.

| **Table 1**  *Confirmatory Factor Analysis for Male and Female Participants in Study 3* | | | | | |
| --- | --- | --- | --- | --- | --- |
| Group | Chi-square Test of Exact Fit | CFI | TLI | RMSEA | SRMR |
| Male | X^2^(31) = 112.91, p < .05 | 0.96 | 0.94 | 0.09 | 0.03 |
| Female | X^2^(31) = 139.40, p < .05 | 0.97 | 0.96 | 0.06 | 0.03 |

Overall, we found tentative evidence for configural, metric, and scalar invariance (Table 2). Although the chi-square difference test indicated that the metric and scalar invariance models were significantly different from each other, the degree of change in the other fit indices were negligible. In particular, we observed less than 0.01 unit decrease in the CFI between measurement models, satisfying he most commonly used criterion for assessing measurement invariance (Cheung & Resvold, 2002; Borstein, 2016). Taken together, this suggests that the factor structure, factor loadings, and item intercepts for the UBC-SSCS are comparable for male and female participants in our sample.

| **Table 2**  Measurement Invariance Models | | | | | | | | | |
| --- | --- | --- | --- | --- | --- | --- | --- | --- | --- |
| Model | Model Fit | | | | | Model Difference | | | |
|  | X^2^ | CFI | TLI | RMSEA | SRMR | △M | △df | △X^2^ | *p* |
| M1: Configural | X^2^(62) = 252.31, *p* < .05 | 0.968 | 0.953 | 0.071 | 0.030 | - | - | - | - |
| M2: Metric | X^2^(76) = 265.38, p < .05 | 0.968 | 0.962 | 0.064 | 0.042 | M2 – M1 | 14 | 13.07 | .52 |
| M3: Scalar | X^2^(84) = 295.35, p < .05 | 0.964 | 0.962 | 0.064 | 0.044 | M3 – M2 | 8 | 29.96 | <.001 |
